# Brain Metastasis in Pancreatic Cancer

**DOI:** 10.3390/ijms14024163

**Published:** 2013-02-19

**Authors:** Johannes Lemke, Jan Scheele, Thomas Kapapa, Christian Rainer Wirtz, Doris Henne-Bruns, Marko Kornmann

**Affiliations:** 1 Clinic of General and Visceral Surgery, University Hospital Ulm, Albert-Einstein-Allee 23, Ulm 89071, Germany; E-Mails: johanneslemke@gmx.de (J.L.); jan.scheele@uniklinik-ulm.de (J.S.); doris.henne-bruns@uniklinik-ulm.de (D.H.-B.); 2 Clinic of Neurosurgery, University Hospital Ulm, Albert-Einstein-Allee 23, Ulm 89071, Germany; E-Mails: thomas.kapapa@uniklinik-ulm.de (T.K.); rainer.wirtz@uniklinik-ulm.de (C.R.W.)

**Keywords:** pancreatic cancer, pancreatic carcinoma, surgery, brain metastasis

## Abstract

Pancreatic cancer is a fatal disease with a 5-year survival rate below 5%. Most patients are diagnosed at an advanced tumor stage and existence of distant metastases. However, involvement of the central nervous system is rare in pancreatic cancer. We retrospectively analyzed all cases of brain metastases in pancreatic cancer reported to date focusing on patient characteristics, clinical appearance, therapy and survival. Including our own, 12 cases of brain metastases originating from pancreatic cancer were identified. In three patients brain metastases were the first manifestation of pancreatic cancer. All other patients developed brain metastases during their clinical course. In most cases, the disease progressed rapidly and the patients died within weeks or months. However, two patients showed long-term survival. Of note, both patients received resection of the pancreatic cancer as well as curative resection of the metachronous brain metastases. Brain metastases in pancreatic cancer are a rare condition and usually predict a very poor prognosis. However, there is evidence that resection of brain metastases of pancreatic cancer can be immensely beneficial to patient’s survival, even with the chance for cure. Therefore, a surgical approach in metastatic pancreatic cancer should be considered in selective cases.

## 1. Introduction

Around 44,000 new cases of pancreatic cancer are estimated to have occurred in the US in 2012, a relatively rare cancer entity compared to other malignancies of the gastrointestinal tract [1]. However, pancreatic cancer, in most cases a ductal adenocarcinoma, is one of the major challenges in oncology evidenced by the fact that the 5-year survival rate among pancreatic cancer patients is below 5% [2]. Of note, the survival rate has not changed during recent decades, despite tremendous advances in cancer diagnostic and therapy. This can be attributed to the fact that the only chance for cure is the complete surgical resection of the tumor, which, however, can only be performed in about 20% of all patients diagnosed [2]. The remaining patients receive palliative treatment and in these patients median survival drops below six months [3]. Three major factors are responsible for these alarming figures. Early diagnosis of pancreatic cancer is impeded by the fact that specific symptoms of early disease are often absent or unspecific. Secondly, the causes for development of pancreatic cancer are largely unknown. This fact hindered the identification of risk groups and therefore the establishment of preventive screenings for pancreatic cancer, which have been successfully established for other malignancies like colorectal and breast cancer [4,5]. Thirdly, and probably most importantly, pancreatic cancer is a highly aggressive malignancy with invasive and metastasizing properties. It shows resistance to most conventional therapeutic regimes, presumably due to its oncogenic background.

If cancer-directed surgery with curative intention is considered in pancreatic cancer, in general, partial pancreaticoduodenectomy is performed for tumors of the pancreatic head. For tumors of the pancreatic corpus or tail, left pancreatectomy is the surgical approach of choice. In patients presenting with advanced disease, palliative treatment comprises chemotherapy, radiotherapy and palliative surgery in symptomatic patients [2]. Importantly, it has been shown that resection of advanced pancreatic cancer does not increase survival, however, does impair quality of life [6]. Therefore, pre-operative staging is crucial to decide whether a patient will benefit from surgical resection and whether the resection is feasible from the surgical point of view. Two criteria usually define inoperable pancreatic cancer: Firstly, a locally advanced tumor with infiltration of the superior mesenteric artery so that complete resection cannot be achieved (locally unresectable disease) and secondly, the existence of synchronous distant metastasis (systemic disease). Like other cancers of the gastrointestinal tract which are drained by the portal vein pancreatic cancer metastasizes preferentially to the liver. In more than 80% of all pancreatic cancer patients with distant metastasis, cancer spread to the liver is detected. Lung and bone metastases follow, but to a much lesser extent [7]. Existence of distant metastases has been shown to impair survival to a median of 2.5 months [3]. Although liver metastases are most frequent in pancreatic cancer, hematogenous spreading of pancreatic cancer to many sites has been reported, including bone, skin, kidneys, thyroid gland, intestine, heart, testis and brain [7].

Brain metastases are a major challenge in cancer therapy due to the fact that about 15%–30% of all cancer patients will develop brain metastases in the course of their disease [8]. Of note, brain metastases are more common than primary brain tumors [9]. However, the vast majority of brain metastases arise from only three malignancies: lung cancer, breast cancer and malignant melanoma. Although most brain metastases originate from lung cancer (~50%), melanoma patients exhibit the highest probability to develop brain metastasis [10]. Importantly, brain metastases are associated with a very poor prognosis. The median survival of randomized controlled trials is mostly in the range of weeks or months, cured patients are subject to case reports [11]. Despite being a major feature of metastatic cancer in general, brain metastases are extremely rare in gastrointestinal cancer. The incidence of brain metastasis is about 4% in colorectal and below 1% in gastric cancer [12–14]. In both cases brain metastases are known to predict a very poor survival [15,16]. Moreover, brain metastases are extremely rare in pancreatic cancer: Only a few cases of this unusually metastatic site of pancreatic cancer have been reported so far. Here, we systematically reviewed all reported cases of brain metastases originating from pancreatic cancer focusing on patient characteristics, clinical appearance, therapy and outcome.

## 2. Brain Metastasis in Pancreatic Cancer

In pancreatic cancer the majority of patients are diagnosed with an already advanced, metastatic disease appointing its dismal prognosis. Despite being a very metastatic cancer, involvement of the nervous system has been shown to be extremely rare in pancreatic cancer. Our literature search revealed only 12 reports of patients diagnosed with brain metastases originated from pancreatic cancer ante-mortem. These cases are summarized in Table 1.

In 1990, Kuratsu and colleagues reported the first two cases of brain metastases in pancreatic cancer in English literature [17]. The first patient, a 56-year old man underwent surgical resection for adenocarcinoma of the pancreatic head. One year later he presented with right hemiparesis. Radiology and subsequent biopsy revealed a supraclavicular lymph node metastasis as well as a metastasis of the right thalamus without evidence of further tumor progression. The lymph node metastasis was resected and the brain metastasis was treated with an intraventricular catheter (Ommaya reservoir), Neocarzonstatais injection and radiotherapy under which the symptoms attenuated. However, the patient died nine months later of pneumonia. Whether his death was related to tumor progression was not reported [17]. The second patient described by these authors was a 58-year old male who developed dizziness and headache five months after a pancreatic cancer had been diagnosed. As synchronous liver metastases had been detected at point of diagnosis, the patient underwent palliative treatment without primary tumor resection. As correlate for his neurological symptoms a metastasis of the cerebellum as well as meningeal carcinomatosis were diagnosed. The solitary brain metastasis was resected, however the patient died within two weeks [17].

In 2001, Ferreira Filho and colleagues described the unique case of a 49-year old man with meningeal carcinomatosis without solid brain metastases. The patient had been diagnosed with metastatic pancreatic cancer for which he underwent palliative chemotherapy. One month later the patient developed headache and vomiting. Remarkably, the performed CT-scan was without any pathological findings. However, a performed lumbar punction and subsequent histological examination showed enrichment of neoplastic cells which originated from the known pancreatic cancer. The palliative treatment was extended to intrathecal chemotherapy. However, the neurological symptoms progressed and the patient died six weeks later [18].

Kamar and co-workers described a patient who was diagnosed with metastatic pancreatic cancer and received palliative chemotherapy. Half a year later the patient suffered from various neurological symptoms. Diagnostics revealed multiple brain metastases. The neurological symptoms rapidly progressed and the patient died within two weeks [19].

In 2003 a large retrospective study of nervous system involvement in pancreatic cancer was published [20]. In their study, the authors enrolled 1229 patients diagnosed with pancreatic cancer between 1980 and 2000. In line with the fact that to this point only a handful cases of brain metastases originating from pancreatic cancer were reported, the authors found brain metastases in only four of 1229 patients (0.33%). All four patients were male (48–62 years old) and presented with typical neurological symptoms. In two patients, who received palliative therapy for a previously diagnosed metastatic pancreatic cancer, brain metastases were detected in follow-up examinations 4 and 5 months after diagnosis, respectively. Both patients received radiation of the brain but died shortly after. Interestingly, in the two other patients, the neurological symptoms arising from the brain metastases were the first manifestation of a pancreatic carcinoma, which could be subsequently diagnosed as the primary site of the metastases. In addition to the synchronous brain metastasis both patients had further distant metastasis. Both patients did not receive any treatment, symptoms rapidly progressed and the patients passed away [20].

Caricato *et al.* reported on a patient who developed a cerebellar metastasis two years after curative surgical resection of a pancreatic cancer. Initially, the brain metastasis could be successfully resected. However, the patient developed multiple, recurrent brain metastases and subsequently underwent palliative chemotherapy. The final outcome of this case was not reported [21].

We recently described two further patients developing brain metastases originating from pancreatic cancer [22]. Both patients (one male, one female) underwent resection of a pancreatic ductal adenocarcinoma of the pancreatic tail with curative intention. Both patients received adjuvant therapy. One patient developed three years after initial diagnosis a solitary liver metastasis which could be successfully resected. In the further course, both patients presented with neurological symptoms and were diagnosed with solitary brain metastases, 11 months and 6 years after initial diagnosis, respectively. Both patients underwent microsurgical resection of the metastases. Follow-up examinations were extended by imaging of the brain. Intriguingly, all symptoms vanished; both patients did not develop any tumor recurrence and enjoy good quality of life, more than 6 and 10 years after diagnosis of the pancreatic tumor.

Very recently, Chiang *et al.* reported a case of a 54-year old male who had been diagnosed with a symptomatic brain tumor which was sub-totally resected. Histological examination indicated a rather metastastic lesion than a primary brain tumor. Subsequent imaging of chest and abdomen then revealed a pancreatic tumor of the uncinate process. Since there was no evidence for further metastasis the tumor was resected. Interestingly, the histological examination revealed an intraductal papillary mucinous neoplasm (IPMN)-derived PDAC. Radiation of the brain and systemic chemotherapy was added. 20 months after diagnosis there was no evidence for tumor recurrence and the sub-totally resected brain metastasis did not show progression [23].

In summary, 12 patients with brain metastases originating from pancreatic cancer have been described between 1990 and 2012. 11 cases (92%) were reported in male and 1 case (8%) was denoted in females. Age ranged from 48 to 67 years. Three patients were diagnosed with synchronous metastasis (25%), all other were diagnosed with metachronous brain metastases (75%). In all cases neurological symptoms, including dizziness, headache, vomiting, nausea and hemiparesis led to the diagnoses of the brain metastases. Three patients (25%) underwent resection of the pancreatic carcinoma and all others (75%) received palliative treatment. The interval between diagnosis of the pancreatic cancer and the detection of the metachronous brain metastasis varied between 2 month and more than 5 years. Five patients (42%) received resection of the brain metastases and all other received best supportive care or palliative treatment consisting of systemic/regional chemotherapy and/or radiotherapy. Eight patients died within one year, most of them within weeks or months. One patient was alive for 20 month after diagnosis and showed stationary status of an incompletely resected brain metastases. The survival of one patient was not reported. Two patients showed long-term survival and are still alive more than 5 years after surgery without signs of tumor recurrence.

## 3. Discussion

Cancer related death is declining for many entities representing recent advances in diagnosis and therapy within the last decades [24]. Despite tremendous efforts, pancreatic cancer remains one of the deadliest cancers, evidenced by the fact that mortality equals its incidence [25]. Its aggressive nature, accompanied by invasive and metastatic properties, can be attributed to its fatal prognosis with a 5-year overall survival below 5% [26]. Complete surgical resection is the only chance for cure [27]. However, less than 20% of all patients are diagnosed at a potentially curative stage and undergo cancer-directed surgery. A non-curative tumor stage in pancreatic cancer is defined by infiltration of the superior mesenteric artery (local unresectability) or by the presence of distant metastases (systemic disease). Of note, the second criteria determines the fate in the majority of patients, as synchronous, distant metastasis are detected in more than two thirds of all patients [3]. These patients undergo palliative treatment and surgical resection of the primary tumor and the metastases is not considered. The most common site of distant metastases in pancreatic cancer is the liver. However, many other sites involved in metastases of pancreatic cancer have been reported. Interestingly, although a common feature in cancer overall, brain metastases appear to be very rare in gastrointestinal cancers [15]. Our study shows that this also applies for pancreatic cancer, as a search in English language literature revealed only 12 patients that were diagnosed ante-mortem with brain metastases originating from pancreatic cancer (Table 1).

The majority of patients diagnosed with pancreatic cancer are older than 65 years [28,29]. Pancreatic cancer patients diagnosed with brain metastases were relatively young with an age between 48 and 68 years. Interestingly, only one of 12 patients was female, all others were male. However, so far, a gender specific effect for incidence or survival in pancreatic cancer has not been observed [28,29]. In this small patient cohort, three patients were diagnosed with synchronous brain metastases which turned out as the first manifestation of a pancreatic cancer. In two of these cases further distant metastases were detected, the patients received palliative treatment and died within four months—in-line with reported survival rates for patients with metastatic disease [3]. Interestingly, Yamada *et al*. reported a case of multiple brain metastases without evidence for a primary tumor despite whole body CT and sonography. However, autopsy revealed a adenocarcinoma of the pancreatic head [30]. Of note, also this patient showed multiple metastatic lesions at different sites.

Of all patients, nine were diagnosed with metachronous brain metastasis. The interval between diagnosis of pancreatic cancer and the detected brain metastases varied between two months up to more than six years. Until recently, in all so far reported cases of brain metastases originating from pancreatic cancer, the systemic disease progressed rapidly and all patients died shortly after diagnosis or entered palliative treatment. Based on these observations, brain metastases associated with pancreatic cancer predicted a fatal course of an already poor overall-prognosis. However, we recently added two patients to the list of cases diagnosed with brain metastases and pancreatic cancer, who took a different clinical course. Both patients were treated with curative intention for the pancreatic adenocarcinoma as well as for the subsequently occurred brain metastases. Intriguingly, both patients showed long-term survival without any evidence for tumor recurrence or any remaining symptoms. To our knowledge this clinical course in unique and differs remarkably to the other cases of this condition described so far. For cancer entities with a higher frequency of brain metastasis (lung and breast cancer) long term survival has been reported after curative resection of brain metastasis [31–33]. However, overall several studies have linked brain metastasis to a very poor prognosis. A large study by Hall and colleagues found a 5-year survival rate of only 2.5% in case of metastatic brain tumors [34] underlining the curiosity of long-term survivors.

As outlined above, metastatic pancreatic cancer is in general considered as palliative and cancer-directed surgery is not considered. Some authors reported the resection of metachronous and synchronous liver metastasis as a safe therapeutic option with potential benefit in selected patients. Despite these rare reports, resection of synchronous or metachronous (liver) metastasis originating from pancreatic cancer is still discussed controversially [35–40]. However, for another cancer entity of the gastrointestinal tract, colorectal cancer, resection of liver metastasis has been established as standard therapy with chance for cure [41]. Furthermore, also for gastric cancer, aggressive treatment regimes, including resection of liver metastases, have provided encouraging results [42]. Based on the very few reports on brain metastases in pancreatic cancer, it is hard to predict the factors that determine which patient might benefit from aggressive treatment, including resection of distant metastases. Both patients who achieved long-term survival were initially treated with cancer-directed surgery with curative intention for the pancreatic neoplasm. Most other patients with brain metastasis were treated with palliative intention due to advanced initial tumor stage. In addition, both survivors had single solitary metachronous brain metastasis for which a complete (R0) resection could be achieved. Interestingly, in both patients the pancreatic tumor was located in the pancreatic tail, although the incidence of head carcinomas is a lot higher than those located in the tail or corpus. Although it has been reported that the overall-survival is impaired in pancreatic tail cancers, the same report also found that early staged pancreatic tail tumors showed an improved survival compared to early staged head tumors [43]. This survival benefit in a small subset of pancreatic cancer patients could apply for the two long-term survivors identified in this study. In addition, in both cases the interval between diagnosis of the primary tumor and detection of the brain metastases was relatively long compared to other reported cases with a fatal clinical course. Of note, in both cases, histological examination of the metastasis confirmed a metastasis of the pancreatic ductal adenocarcinoma [22]. A recent report described a patient with a solitary brain metastasis which originated from pancreatic cancer derived from an oncocytic-type intraductal papillary mucinous neoplasm (IPMN) [23]. This patient was still alive 20 months after surgery. Although our own two cases were described as ductal adenocarcinomas without any evidence of an IPMN background, we cannot exclude that these metastasized cases of pancreatic cancer with a favorable prognosis may have a specific genetic profile or similar precursor lesions. A recent study suggested that the prognosis of ductal adenocarcinomas of the pancreas may depend on the precursor epithelial subtypes [44].

## 4. Materials and Methods

A Medline query was performed for the terms “pancreatic cancer”, “pancreatic carcinoma” and “brain metastases” to identify all cases known to the literature. All English literature was selected for further analysis. Data were collected and analyzed retrospectively according to patient characteristics, clinical presentation, therapy and survival.

## 5. Conclusions

In summary we conclude that brain metastases originating from pancreatic cancer are rare and usually associated with impaired survival. They are often associated with a locally advanced primary tumor as well as the existence of further distant metastases at other sites. In the majority of the reviewed cases, disease progressed rapidly and the patients died soon under palliative treatment. However, we recently reported on two patients that underwent curative resection for primary pancreatic cancer and developed metachronous, symptomatic brain metastasis. Metastases were completely resected and patients showed long-term survival with no signs of tumor recurrence. These cases emphasize that resection of distant (brain) metastasis can result in enormous benefit, even with a chance for cure. Therefore, a surgical approach of metachronous metastatic lesions of pancreatic cancer should be considered as a treatment strategy in selected cases. Evaluation of molecular patterns of such cases of pancreatic cancer with excellent prognosis may be one important future step to learn more about the pathogenesis of this fatal disease.

## Figures and Tables

**Table 1:** Reported cases of brain metastases in pancreatic cancer.

Author (reference)	Age/sex	PC-therapy	Metastases	Localization/histology	Symptoms	BM: number/localisation	BM-treatment	Interval (months)	Survival (months)
Kuratsu [17]	56/m	curative	LN	Head/AC	Hemiparesis	Single/Thalamus	Radiation Chemo	12	9
Karatsu [17]	58/m	palliative	Liver	NR/AC	Dizziness Headache	Single/Cerebellum	Resection	5	1
Ferreira [18]	49/m	palliative	Bone	NR/AC	Headache Vomiting	Carcinomatous meningitis	Chemo	5	2
El Kamar [19]	56/m	palliative	Liver	Tail/AC	Hemiparesis	Multiple/Cerebrum and Pons	-	6	1
Park [20]	48/m	palliative	Lung	NR/AC	Headache Hemiparesis	Multiple/-	Radiation	4	4
Park [20]	52/m	palliative	Liver	NR/AC	Hemiparesis Aphasia Behaviour	Single/Cerebrum	Radiation	5	4
Park [20]	51/m	palliative	Liver Lung Bone	NR/AC	Headache Aphasia Hemiparesis	Single/Cerebrum	-	0	4
Park [20]	62/m	palliative	Lung	NR/AC	Hemiparesis	Multiple/Cerebrum	-	0	4
Caricato [21]	67/m	curative	-	Head/AC	Vomiting		Resection Chemo	24	12 *
Lemke [22]	48/w	curative	Liver	Tail/AC	Headache Vomiting Nausea	Single/Cerebellum	Resection Radiation	64	>10 years *
Lemke [22]	66/m	curative	-	Tail/AC	Headache Nausea Hemiparesis	Single/Cerebrum	Resection Radiation	11	>6 years *
Chiang [23]	54/m	curative	-	Uncinate Process/AC	Hemiparesis	Single/Cerebrum	Resection Radiation Chemo	0	20 *

PC-therapy: therapy performed for pancreatic cancer; Metastases: distant metastases detected at initial diagnosis; Localization/histology: localization of pancreatic cancer, histology of pancreatic cancer (AC = adenocarcinoma); BM: number/localization: Number of detected brain metastases (single/multiple) and localization; BM-therapy: therapy performed for brain metastases; Interval: interval between diagnosis of pancreatic cancer and appearance of brain metastases; Survival: reported survival after diagnosis of brain metastases;

* patient alive at time of report.
